# Human thymic Treg-derived extracellular vesicles display a defined proteomic and miRNA signature with donor-variable suppressive activity

**DOI:** 10.1186/s10020-026-01576-4

**Published:** 2026-08-06

**Authors:** Marta Fernández-Castillo, Elena Blázquez-López, Esther Bernaldo-de-Quiros, Marta Martínez-Bonet, Jorge Gallego-Valle, Ana Pita, Javier Vaquero, Perceval Vellosillo, Rafael Correa-Rocha, Marjorie Pion

**Affiliations:** 1 Laboratory of Immune-Regulation (LIR), Gregorio Marañón Health Research Institute (IiSGM), Hospital General Gregorio Marañon, C/Dr. Esquerdo, 46, 28007 Madrid, Spain; 2 HepatoGastro Lab, Instituto de Investigación Sanitaria Gregorio Marañón (IiSGM), 28007 Madrid, Spain; 3 Group of Advanced Immuno-Regulation (GIRA), Gregorio Marañón Health Research Institute (IiSGM), Hospital General Gregorio Marañon, C/Dr. Esquerdo, 46, 28007 Madrid, Spain; 4 Paediatric Cardiac Surgery Unit of Gregorio, Marañón University Hospital (HGUGM), C/Dr. Esquerdo, 46, 28007 Madrid, Spain; 5 Centro de Investigación Biomédica en Red de Enfermedades Hepáticas y Digestivas (CIBEREHD), Madrid, Spain; 6 Unidad de Investigación Materno Infantil Fundación Familia Alonso (UDIMIFFA), Gregorio Marañón Health Research Institute (IiSGM), Madrid, Spain

**Keywords:** Extracellular vesicles, Regulatory T cells, Immunosuppression, Proteomic, Transcriptomic

## Abstract

**Background:**

Extracellular vesicles (EVs) are key mediators of intercellular communication that transfer functional molecules reflective of their parent cells and are increasingly explored as cell-free therapeutic agents. Immunoregulatory cell types, including regulatory T cells (Tregs), produce EVs with potential anti-inflammatory properties. While thymus-derived Tregs (thyTregs) exhibit high stability and potent suppressive activity, their capacity to generate functional EVs remains unknown.

**Methods:**

EVs were isolated by size exclusion chromatography or by immunocapture from ex vivo–expanded human thyTregs and characterized by electron microscopy, nanoparticle tracking analysis, and Western blotting. Proteomic and miRNA transcriptomic analyses were performed to define EV cargo. Functional activity was assessed by co-culturing EVs with activated allogeneic peripheral blood lymphocytes (PBLs) and evaluating proliferation, activation markers, and cytokine production.

**Results:**

Tetraspanin-positive ThyTreg-derived EVs exhibited typical vesicular morphology, a mean size of ~ 200 nm, and expression of canonical EV markers (CD9, TSG101). Proteomic profiling identified 314 proteins enriched in immune-related pathways, including CD25, CTLA-4, TGF-β1, ICOS, and granzyme B, reflecting the parental Treg phenotype. Transcriptomic analysis revealed a distinct miRNA signature dominated by let-7 family members and miR-148a-3p, with predicted targets involved in TGF-β/BMP and MAPK signaling. Functionally, thyTreg-EVs suppressed CD4⁺ and CD8⁺ T cell proliferation in a dose-dependent but heterogeneous manner and, in some donors, induced responder cell death. No significant changes in activation marker expression or cytokine production were observed.

**Conclusions:**

Human thyTregs release EVs with immunosuppressive properties and a defined molecular cargo. These findings identify thyTreg-derived EVs as a novel, cell-free platform with potential for immunomodulatory therapies.

**Supplementary Information:**

The online version contains supplementary material available at https://doi.org/10.1186/s10020-026-01576-4.

## Background

Regulatory T cells, characterized by the expression of the alpha chain interleukin-2 receptor (CD25) and the transcription factor forkhead box P3 (FOXP3), are crucial immune regulators explored as therapies for autoimmune diseases, infections, and transplant complications (Lu et al. 2017). Preclinical studies showed strong benefits (Sagoo et al. 2011; Sánchez-Fueyo et al. 2020), and human trials with peripheral Tregs (pTregs), confirm their safety and some efficacy, especially in graft-versus-host disease (GvHD) and type 1 diabetes (Trzonkowski et al. 2009; Di Ianni et al. 2011; Marek-Trzonkowska et al. 2012). However, adult pTreg therapies face limitations due to reduced function, contamination, and instability after expansion, particularly in patients with autoimmune disease (Hoffmann et al. 2009). To address this, cell therapies based on thymus-derived Tregs (thyTregs) have been developed, offering superior stability and function with minimal manipulation (Bernaldo-de-Quirós et al. 2022). Early clinical trial results in pediatric heart transplant patients suggest thyTregs are safe and potentially effective (NCT04924491, (Bernaldo-de-Quirós et al. 2023)).

Tregs suppress immune responses through diverse mechanisms. These include interleukine 2 (IL-2) consumption via high CD25 expression, secretion of immunosuppressive cytokines (transforming growth factor-beta (TGF-β), IL-10, and IL-35 (Liu et al. 2008)), generation of adenosine through CD39/CD73 (Antonioli et al. 2013), and expression of checkpoint receptors (cytotoxic T-lymphocyte associated protein 4 (CTLA-4), programmed cell death protein 1 (PD-1), lymphocyte-activation gene 3 (LAG-3), and T cell immunoreceptor with Ig and ITIM domains (TIGIT)) (Li et al. 2020). They can also kill target cells with granzymes and perforins (Gondek et al. 2005), inhibit dendritic cell antigen presentation (Akkaya et al. 2019), and communicate via extracellular vesicles (EVs) (Yang et al. 2024; Tung et al. 2020).

EVs are diverse, membrane-bound structures that carry lipids, proteins, and nucleic acids, enabling cell-to-cell communication without direct contact. Tregs produce EVs, likely upon T cell receptor (TCR) activation (Asemani et al. 2022), and these vesicles exhibit immunosuppressive effects similar to Tregs themselves (Aiello et al. 2017; Chen et al. 2019). Their function is thought to depend on cargo such as CD73, which degrades ATP (Smyth et al. 2013), and specific miRNAs (Tung et al. 2020, 2018). However, most of this evidence comes from animal studies. In humans, pTregs have also been shown to release EVs following CD3/CD28 stimulation, although our understanding of EVs derived from human Treg is very limited (Tung et al. 2020; Tao et al. 2025; Thome et al. 2022).

The use of Treg-derived EVs in immunotherapy, rather than the cells themselves could help overcome some limitations of Treg cell-based approaches, such as Treg plasticity and immunogenicity in allogeneic settings (Zhou et al. 2009). While EVs may offer reduced immunogenicity compared to intact allogeneic cells, given their lack of direct antigen-presenting capacity and co-stimulatory machinery, allogeneic EVs are not immunologically inert, and that repeated EV administration can trigger immune clearance (Driedonks et al. 2022). However, encouraged by promising preclinical results, clinical interest in EV-based applications has grown rapidly in recent years (Nazari-Shafti et al. 2021). It remains unknown whether thyTregs produce EVs with immunomodulatory activity, but if so, this would add to their suppressive repertoire.

Here, we show that these thyTreg derived EVs exhibit measurable suppressive activity against activated allogeneic PBLs in vitro. This approach could enable allogeneic treatment strategies for autoimmune diseases and solid organ transplantation.

## Methods

- Thymic Tissue Obtention and Human CD25 + FOXP3 + cell isolation

The study was approved by the Hospital General Universitario Gregorio Marañón (HGUGM) ethics committee (code: TRP4-22–007) and conducted in accordance with the Declaration of Helsinki, with informed consent obtained from legal guardians. Human thymuses discarded during paediatric cardiac surgeries at the Paediatric Cardiac Surgery Unit at the HGUGM were used. thyTregs were isolated as described (Bernaldo-de-Quirós et al. 2022). Briefly, thymic tissue was dissociated in TexMACS GMP medium (Miltenyi Biotec) using the gentleMACS Dissociator (Miltenyi Biotec), and CD25⁺ cells were purified with the Human CD25 MicroBeads II kit (Miltenyi Biotec). Cells (thyTreg day 0) were cultured in TexMACS GMP medium supplemented with 600 U/ml IL-2 (Miltenyi Biotec) and stimulated for 3 days with T Cell TransAct (Miltenyi Biotec) according to the manufacturer’s instructions. T Cell TransAct was selected as the activation reagent because it provides TCR-equivalent CD3/CD28 co-stimulation in a GMP-compatible format ensuring translational relevance of the EV preparations characterized here. Fresh IL-2 was added to the culture medium every 3 days until day 7. After 24 and 48 h after the last cell passage, the cell supernatant containing EVs was collected and subjected to purification protocols (see Sect. 2-c). Concurrently, cells were harvested for viability and phenotypic characterization by flow cytometry (Supplementary Table 1, upper panel). Surface markers were stained first, followed by Fixable Viability Dye eFluor450 (eBioscience). Cells were then fixed and permeabilized using the FOXP3 Transcription Factor Staining Kit (eBioscience) for intracellular labeling. Flow cytometry was performed on a MACSQuant Analyzer 16 (Miltenyi Biotec), and data were analyzed with Kaluza software (version 2.1, Beckman Coulter).

(b) Peripheral blood lymphocytes isolation.

Peripheral blood mononuclear cells (PBMCs) were isolated from healthy donor buffy coats provided by the Madrid Transfusion Centre using a Ficoll-Hypaque density gradient (Rafer) and cryopreserved until use. Thawed PBMCs were cultured overnight in complete RPMI 1640 medium with L-glutamine (Lonza), supplemented with 10% fetal bovine serum (FBS) (Biowest), 1% antibiotic mix (125 µg/mL ampicillin, 125 µg/mL cloxacillin, 40 µg/mL gentamicin; Merck Life Science, Madrid, Spain), and 60 U/mL IL-2 (ImmunoTools) to allow monocyte adherence. The following day, peripheral blood lymphocytes (PBLs) were collected, washed, and returned to culture in complete RPMI 1640 medium supplemented with 60 U/mL IL-2. Condition 1 –PBLs were activated with T Cell TransAct for up to 7 days, with fresh IL-2 replenished every 3 days. On day 7, 24–48 h after the last passage, the cell supernatant containing extracellular vesicles (EVs) was collected, and the cells were used as activator cells in immunosuppressive assays (see Sect. 2-f). Condition 2 –PBLs thawed overnight were stained with 2 μM CellTrace Violet (CTVio, Life Technologies) and then stimulated with anti-CD3/CD28-coated beads (Dynabeads, Gibco) at a bead:PBL ratio of 1:2. After 18 h of activation, the beads were removed, and the PBL-CTvio were used as responder cells in suppressive assays (see Sect. 2-f).

(c) EV Purification.

EV-conditioned supernatants (20 mL) were collected from thyTreg cultures at a density of 2 × 10^6^ cells/mL.

Immunocapture: Supernatants (SN) were first cleared by two-step centrifugation at 2,000 × g for 30 min (Soval ST40R, rotor: 75,003,180) and 10,000 × g for 45 min at room temperature (Sorval Legend Micro21R, rotor: 75,003,424, all centrifugations’ steps were done with acceleration: 9, braking: 9). Two milliliters of the cleared supernatant were incubated with the Pan Human EV Isolation Kit (Miltenyi Biotec), which targets the tetraspanins CD9, CD63, and CD81, following the manufacturer’s instructions. EVs were then eluted through a μColumn (Miltenyi Biotec), resulting in 100 μL of elution containing either lysis buffer for Western blotting or isolation buffer for proteomic analysis, depending on the downstream assay.

Size exclusion chromatography (SEC): Supernatants were cleared by centrifugation at 500 × g (Soval ST40R, rotor: 75,003,180) and 10,000 × g (Sorval Legend Micro21R, rotor: 75,003,424) for 10 min at room temperature. A small portion of the total cleared supernatant (SN) was reserved, and the remaining SN was concentrated using a Vivaspin 20 filter (Sartorius) at 3,000 × g for 25 min (Sorval Legend Micro21R, rotor: 75,003,424, all centrifugations’ steps were done with acceleration: 9, braking: 9). The concentrated sample was applied to a qEVsingle 70 nm Gen 2 column (Izon Sciences), and thirty 170 μL fractions were eluted with filtered PBS. Protein concentration in each fraction was determined using the Bradford Plus Protein Assay Kit (Pierce) according to the manufacturer’s instructions. Fractions 6–9 containing EVs, and fractions 20–23, containing soluble proteins were pooled, quantified by Nanoparticle Tracking Analysis (NTA), and stored at 4 °C for up to 48 h before use. Thus, three distinct fractions were obtained from the cultured cell supernatants: the total cleared supernatant before concentration (SN), the EV fraction, and the protein fraction. The NTA evaluation of the samples was based on the analysis of three 60-s videos at an infusion rate of 40 (arbitrary units) on a Nanosight NS300 (Malvern Instruments, Worcestershire, UK) using the software NTA v3.4 Build 3.4.003 (Malvern Panalytical Ltd.). Samples were analyzed under standard acquisition settings using a screen gain of 1–10 and camera level of 13. Particle tracking was performed using a detection threshold of 15–28, adjusted per sample to optimize particle detection. The observed distribution, with a peak around ~ 200 nm, reflects the hydrodynamic diameter of the isolated EVs. NTA has a lower limit of detection of approximately 50–70 nm for biological vesicles, meaning particles below this threshold are not captured in the measurement. Because measurements were acquired over an extended period during which the instrument underwent maintenance and repair, without external standard, consistent acquisition settings across all runs were not feasible. Consequently, particle concentrations reported here should be interpreted as estimates rather than absolute quantifications.

The isolation strategy employed in this study (SEC fractions 6–9 or immunocapture with anti-CD9/CD63/CD81 beads) selectively enriches for EVs: SEC by hydrodynamic radius (EV-containing fractions collected after the void volume) and immunocapture by tetraspanin surface markers (CD9, CD63, CD81). EVs for functional assays were obtained from 9–13 independent thyTreg donors, depending on the assay, while EVs used for proteomic and miRNA sequencing analyses were obtained from 4 and 8 donors, respectively.

In one experiment, activated 5 × 10^6^ ThyTreg cells were labeled with 2 µM CellTrace Violet (CTVio) and cultured for 48 h. After 48 h, SN were processed by size-exclusion chromatography (SEC) to obtain CTVio-labeled EV fractions, which were used to assess cellular entry. For this, 1 × 10^5^ pre-activated PBLs were treated with different amounts of CTVio-labeled EVs for 1, 4, and 8 h. Cells were then extensively washed and analyzed by flow cytometry for live cells (7-AAD⁻) that were CTVio⁺.

(d) EVs characterization by electron Microscopy (EM)

ThyTreg-EVs isolated by SEC were sent at 4 °C to the National Centre of Electron Microscopy (ICTS-CNME) in Madrid, Spain, for electron microscopy (EM) and image acquisition. Upon receipt, 50 μL aliquots of EVs (4.27 × 10^9^ particles/mL) were placed onto formvar carbon-coated copper grids and negatively stained at room temperature with 2% uranyl acetate. Samples were then imaged at various magnifications using a JEOL JEM-1400 microscope (JEOL Ltd) operating at 100 kV and equipped with a CCD Gatan SC Orius 200 camera (Gatan, Ametek).

(e) EVs characterization by Western Blotting

ThyTreg-EVs isolated by immunocapture were lysed in RIPA buffer supplemented with protease inhibitors (Thermo Fisher Scientific). Protein concentration was determined using the Bradford Plus Protein Assay Kit (Pierce) according to the manufacturer’s instructions. Fifty micrograms of denatured protein were separated on a 12% polyacrylamide gel (BioRad) and transferred onto a PVDF membrane (Merck). The membrane was incubated with rabbit anti-human CD9 antibody (1:2,000; Abcam) and mouse anti-human Tumor Susceptor Gene 101 (TSG101) antibody (1:200; Santa Cruz Biotechnology). Detection was performed using horseradish peroxidase (HRP)-conjugated secondary antibodies: donkey anti-rabbit IgG (1:5,000; GE Healthcare) and sheep anti-mouse IgG (1:2,000; GE Healthcare). Protein bands were visualized with a chemiluminescence detection kit (BioRad) and imaged using the Alliance 4.7 System (Uvitec).

(f) Suppression Assays with EVs

A total of 1 × 10^5^ preactivated responder PBL-CTvio were co-cultured with varying amounts of thyTreg- or PBL-derived EVs (10^9^, 10^8^, or 10^7^ particles) in complete RPMI 1640 medium supplemented with 60 U/mL IL-2. CTVio-labeled PBLs cultured alone in the presence or absence of anti-CD3/CD28 beads served as positive (CT⁺) and negative (CT⁻) controls of proliferation, respectively. In the CT⁺ condition, filtered EV-free PBS was added at a volume equivalent to that used for treatment with 10⁹ EVs. After 3 days of culture at 37 °C and 5% CO₂, co-culture supernatants were collected and stored at − 80 °C for cytokine analysis. Cells were harvested and stained for flow cytometry using a MACSQuant Analyzer 16 and analyzed with Kaluza software (Supplementary Table 1, lower panel). 7-Aminoactinomycin D (7-AAD; Sigma-Aldrich) was used to discriminate live from dead cells. Suppression of proliferation was calculated as the percentage reduction relative to activated PBLs cultured alone, using the Division Index method (McMurchy and Levings 2012).

(g) Suppression Assays with thyTreg cells and PBL cells

Non-activated PBL-CTvio were co-cultured with allogeneic thyTregs or PBLs (previously activated for 7 days) at various cell-to-PBL ratios (1:1, 1:2, 1:4, and 1:8) in the presence of anti-CD3/CD28-coated beads at a bead-to-PBL ratio of 1:2. Cultures were maintained in X-VIVO 15 medium (Lonza) supplemented with 5% human AB serum and 600 U/mL IL-2. PBLs cultured alone, in the presence or absence of anti-CD3/CD28 beads, served as positive (CT⁺) and negative (CT⁻) proliferation controls, respectively (Supplemental Fig. 1). Viability and suppression of proliferation of responder CTVio-CD4⁺ and CD8⁺ T cells were evaluated following the workflow described in Sect. 2-f.

**Fig. 1:**
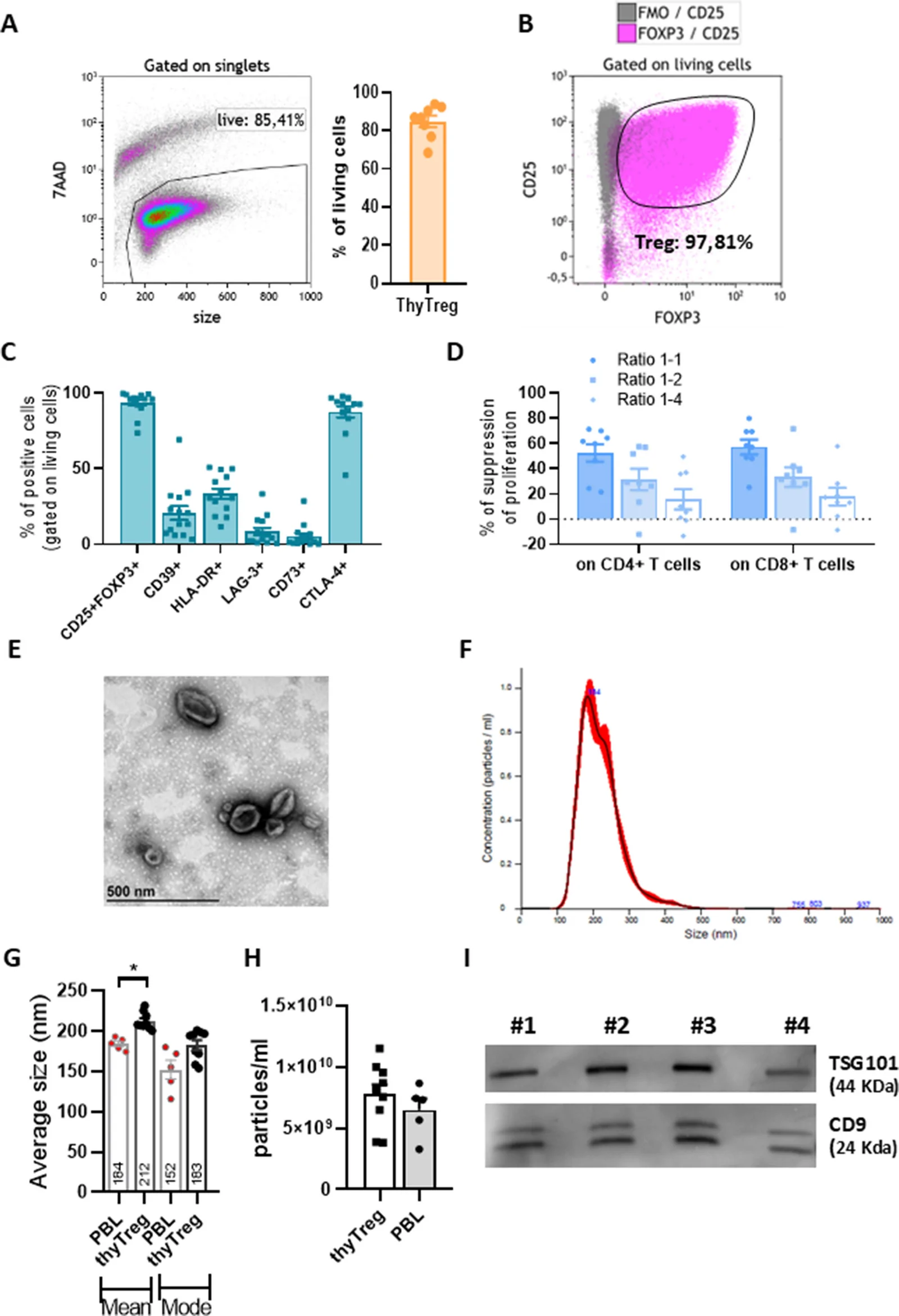
Characteristics of thyTreg cells and EV-derived thyTreg and PBLs.** A** Cell viability. Representative dot plot of viability assessed by 7-AAD staining (left) and histogram of viability frequencies (right; *N* = 8, mean ± SEM). **B** Classical Treg markers. Overlay dot plot of thyTregs stained for FOXP3 and CD25 (pink) versus FOXP3 FMO control (gray), gated on live cells. **C** ThyTreg phenotype. Frequency of CD25⁺FOXP3⁺, CD39⁺, HLA-DR⁺, LAG-3⁺, CD73⁺, and CTLA-4⁺, gated on live cells (*N* = 14, mean ± SEM). **D** Suppressive capacity. CTVio-labeled PBLs were co-cultured with thyTregs at 1:1, 2:1, and 4:1 ratios. Suppression of CD4⁺ and CD8⁺ responder T-cell proliferation was calculated using the division index method (Welsh et al. 2024) (*N* = 8, mean ± SEM). **E** Transmission Electron Microscopy (TEM). Representative images showing morphology of thyTreg-particles isolated from SEC fractions 6–9. **F** Size and concentration of ThyTreg-particles. Representative nanoparticle tracking analysis (NTA) profile of particle size distribution from pooled particles-enriched SEC fractions (Marek-Trzonkowska et al.; Hoffmann et al.; Bernaldo-de-Quirós et al. **G** Particle size distribution. Histogram of mean and mode particle sizes from pooled particle-enriched SEC fractions (6–9, from PBL and thyTreg culture supernatants (*N* = 5 for PBL-particles; *N* = 9 for thyTreg-particles; mean or mode ± SEM). **H** Particle concentration. Histogram showing particle concentration in pooled SEC fractions 6–9 obtained from 20 mL of PBL (*N* = 5 independent donors) or thyTreg (*N* = 9 independent donors) culture supernatants (mean particles/mL ± SEM). **I** ThyTreg-EV markers. Western blot analysis of canonical EV markers TSG101 (44 kDa) and CD9 (24 kDa) in four independent ThyTreg-EV samples isolated by immunocapture (anti-CD9/CD63/CD81 beads)

(h) Proteomic Analysis by Mass Spectrometry

ThyTreg-derived EVs isolated by immunocapture were sent for proteomic analysis at the Proteomics Unit of the Complutense University of Madrid (Madrid, Spain). Samples were dried, digested with Trypsin-LysC using the iST BTC kit (PREOMICS), and peptides were eluted, dried, reconstituted in LC-LOAD buffer, and quantified by Qubit (Thermo Fisher Scientific). For Liquid Chromatography–Mass Spectrometry (LC–MS/MS), 1 μg of peptides was analyzed using a Vanquish Neo nano-HPLC system coupled to a Q-Exactive HF mass spectrometer (Thermo Scientific). Peptides were separated on a C18 reversed-phase column and ionized via nanoelectrospray. Data were acquired in full-scan MS mode at 60,000 resolution and MS/MS was performed using data-dependent acquisition (DDA) with up to 15 precursors selected per microscan, fragmented by high-energy collision dissociation (HCD). Proteins were identified using Proteome Discoverer 3.0 (Thermo Scientific) and Mascot 2.8 (www.matrixscience.com) against the Uniprot SP-Human database and a contaminants database (containing 20,423 sequences and 247 sequences of the most common contaminants as of date 8/11/23) from Uniprot (https://www.uniprot.org/). Peptides and proteins were considered confidently identified with the false discovery rate (FDR) < 1%, at least one unique peptide (CI > 99%), and further filtered by a q-value < 0.01 for Peptide-Spectrum Matches (PSMs). Relative protein abundance was estimated using the Normalized Spectral Abundance Factor (NSAF, Supplemental Table 2). The approach to determine the relative abundance of proteins based on the estimation of the NSAF index (McIlwain et al. 2012) allows for comparison of protein abundance across one or more samples based on the number of spectra assigned to each protein for identification, normalized by the protein size to allow intra-sample and inter-sample comparisons.

(i) RNA extraction, Real-time PCR and microarray

Total RNA from thyTreg cells was extracted using the RNeasy Mini Kit (Qiagen). Reverse transcription was performed on 300 ng of RNA per sample using the miRCURY LNA RT Kit (Qiagen) in a SimpliAmp thermal cycler (Applied Biosystems). Quantitative real-time PCR (qPCR) was conducted with the resulting cDNA diluted 1:60 using the miRCURY LNA SYBR Green PCR Kit (Qiagen) and specific miRNA primer mixes listed as follow, cel-miR-39-3p, hsa-miR-21-5p, hsa-miR-143-3p, hsa-miR-148a-3p, hsa-miR-26a-5p, hsa-let-7f-5p, hsa-miR-122-5p, hsa-let-7i-5p, hsa-miR-451a (all from Qiagen, references YP00203952, YP00204230, YP00205992, YP00205867, YP00206023, YP00204359, YP00205664, YP00204394, YP02119305, respectively). qPCR was performed on a QuantStudio 3 system (Applied Biosystems), and expression levels were quantified using the standard curve method generated from miRNA mimics. The following miRNA mimics were used for standard curve generation: miRNA control (Ref: MIH00000), hsa-miR-21-5p (Ref: MCH01562), hsa-miR-143-3p (Ref: MCH01315), hsa-miR-148a-3p (Ref: MCH01337), hsa-miR-26a-5p (Ref: MCH01631), hsa-let-7f-5p (Ref: MCH01019), hsa-miR-451a (produced on demand), hsa-miR-122-5p (Ref: MHC01089), and hsa-let-7i-5p (Ref: MCH01025), all obtained from Condalab. Analysis was carried out using QuantStudio Design & Analysis Software (version 1.5.2).

For thyTreg-EVs isolated by immunocapture, total RNA was extracted using a combined RNeasy Mini Kit (Qiagen) and TRIzol (Invitrogen) protocol, as already described (Prendergast et al. 2018). RNA from eight independent samples was sent to Novogene (Cambridge, UK) for transcriptomic analysis. Sequencing reads were generated from raw Illumina data using CASAVA base calling, then filtered to remove low-quality reads, reads with > 10% undetermined bases (N), primer contamination, poly-nucleotide tails, or lengths outside 18–40 nucleotides. Clean reads were mapped to the reference genome and annotated against miRBase and Rfam. Reads corresponding to rRNA, tRNA, snRNA, snoRNA, and mRNA fragments were removed to identify small RNA sequences.

(j) Functional and Pathway Analysis of Proteins and miRNAs

Protein functional enrichment was performed at the Proteomics Unit of the Complutense University of Madrid using the 'gprofiler2' package in RStudio (Kolberg 2020) with the Ensembl database. Homo sapiens was the reference organism, and terms with *p*-values < 0.01 were considered significant. Databases utilized for biological term enrichment included Gene Ontology (encompassing "Molecular Function" (MF), "Cellular Component" (CC), and "Biological Process" (BP)), Reactome (REAC), WikiPathways (WP), Human Protein Atlas (HPA), and CORUM. KEGG pathway enrichment for 321 proteins was performed using clusterprofiler (Yu et al. 2012) in RStudio with a significance threshold of *p* < 0.05 and FDR correction.

For transcriptomic data from eight thyTreg-EV samples, 430 known and 123 novel miRNAs were detected. Target genes were predicted using miRNATap and miRNAtap.db libraries (Ma et al. 2021) in RStudio, integrating multiple algorithms (DIANA, TargetScan, PicTar, MiRanda and miRDB). Each miRNA-target prediction algorithm assigns a score to interactions, which miRNATap aggregates by geometric mean of the ranrranks which proves to be the most accurate method to generate a final rank indicating the interaction strength. To focus on the most relevant targets, only genes in the top 10% of this ranking (first decile) were selected for further analysis. Functional enrichment of miRNA-associated genes was performed using ClusterProfiler (Xu et al. 2024) library in RStudio, with Gene Ontology (Solinas, Gu-Trantien, Willard-Gallo 2020) and KEGG (Campos-Mora et al. 2022) databases. Clustering analyses were conducted using Euclidean distance in ComplexHeatmap (Gu 2022) library in RStudio, and miRNA-function networks were visualized in Cytoscape (Franz et al. 2023) using the edge-weighted spring embedded layout.

(k) Cytokine Measurements.

Cytokine levels in the supernatants from 3-day PBL co-cultures with thyTreg-EVs were measured using the ELLA Protein-Simple immunoassay (Biotechne) following the kit instructions. Samples were thawed, centrifuged to remove debris, and analyzed in triplicate using SimplePlex cartridges. Results are reported in pg/mL as the mean of replicates. The limit of detection (LOD) and the quantification range for each of the evaluated molecules are as follows: IL-10: 0.08 pg/ml (0.58–2,212 pg/ml); Interferon gamma (IFNγ): 0.04 pg/ml (0.17–4,000 pg/ml); Tumour Necrosis Factor alpha (TNFα): 0.08 pg/ml (0.3–1,160 pg/ml); and IL-6: 0.08 pg/ml (0.28–2,652 pg/ml).

(l) Statistics.

Due to the low sample size, the normality assumption was excluded, and non-parametric tests were used. Statistical significance was determined using one-way ANOVA, Friedman test with Dunn’s multiple comparisons test. Intragroup variability of quantitative variables was expressed as Standard Error of the Mean (SEM). Data was analysed using PRISM software (GraphPad Software version 8.0.1, Inc). In the figure legend, N refers to independent biological replicates (independent donors), not technical replicates.

## Results

(m) General Characteristics of EV-Producing Human thyTreg and Tconv Cells.

After 7 days of activation and expansion, human thyTreg cells showed > 80% viability (84.80 ± 3.03%, % ± SEM, Fig. 1A), though one donor fell below this threshold (~ 65%). and high purity based on FOXP3⁺CD25⁺, gated on living cells (> 90%, 96.23 ± 0.82%, % ± SEM, Fig. 1B), as compared to the fluorescence minus one (FMO) control for the FOXP3 marker (Fig. 1B, grey dots). Marker expression was low for LAG-3 (6.79 ± 3.45%, % ± SEM), CD73 (5.46 ± 2.03%), and CD39 (17.33 ± 7.36%); intermediate for HLA-DR (42.12 ± 5.14%); and high for CTLA-4 (85.78 ± 4.25%, Fig. 1C), as already described (Bernaldo-de-Quirós et al. 2022). Functionally, thyTregs suppressed proliferation of activated CD4⁺ and CD8⁺ T cells in a dose-dependent manner, confirming their regulatory activity (Fig. 1D, Supplemental Fig. 1). EVs further used in this study were therefore derived from phenotypically and functionally validated thyTregs.

As a control, activated PBLs displayed high viability but low FOXP3⁺CD25⁺ expression (Supplemental Fig. 2A-C) and lacked suppressive capacity (Supplemental Fig. 2D), as expected.

(n) Characteristics of EVs Derived from Human thyTreg and Tconv Cells.

SEC was performed on supernatants from activated and expanded thyTregs or PBLs. Fractions 6–9, enriched in EVs, and fractions 20–23, enriched in proteins, were collected according to manufacturer’s guidance. Electron microscopy revealed the typical cup-shaped morphology of thyTreg-derived EVs from fractions 6–9 (Fig. 1E).

NTA showed thyTreg-particles had a mean size of 212.3 ± 3.5 nm and a mode of 182.8 ± 6.1 nm (nm ± SEM, Fig. 1F-G). PBL-particles were significantly smaller, with a mean of 184.1 ± 3.4 nm and mode of 152.2 ± 11.6 nm (nm ± SEM, Fig. 1G). In terms of concentration, thyTreg-particles showed an NTA-derived estimated particle concentration of 7.5 × 10⁹ ± 8.3 × 10⁸ particles/ml (particles/ml ± SEM; range: 3.8 × 10⁹–1.15 × 10^1^⁰, Fig. 1H), while PBL-particles reached 5.5 × 10⁹ ± 9.2 × 10⁸ particles/ml (range: 3.3 × 10⁹–8.7 × 10⁹). Western blot confirmed the presence of canonical EV markers CD9 and TSG101 on thyTreg-EVs (Fig. 1I). In summary, activated human thyTreg released particles positive for both CD9 and TSG101, which were slightly larger than those produced by activated PBLs.

(o) thyTreg-EVs have suppressive function on allogeneic lymphocytes.

**Fig. 2:**
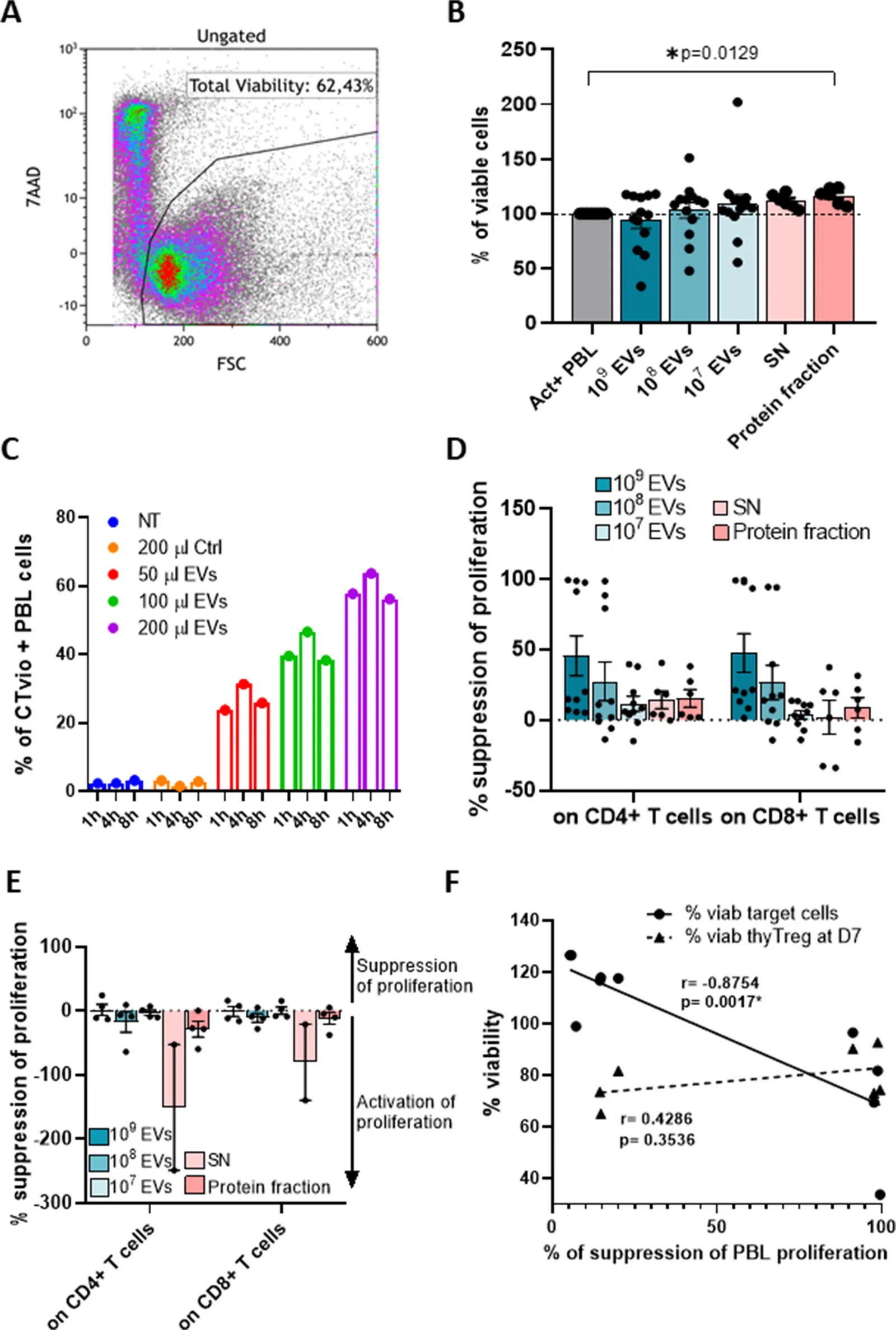
Suppressive effect of EVs on proliferation of allogeneic activated PBLs. CT-Vio-labeled PBLs were pre-activated for 18 h and then treated with three EV doses (10⁹, 10⁸, 10⁷ particles) for 3 days, and analyzed by flow cytometry. Fractions 6–9 (EVs) and 20–23 (proteins) from SEC assay were used. **A** Representative dot plot of viability assessed by 7-AAD staining of CT-Vio-labeled PBLs pre-activated for 18 h and treated with PBS. **B** Cell viability frequencies. Histograms showing viability relative to activated PBLs (Act +). Cell viability was expressed as a percentage relative to the Act + PBS control within each independent experiment (paired normalization), which was set to 100% to account for inter-experiment variability in baseline activation (*N* = 6 for cleared supernatant (SN) and SEC protein fractions (fractions 20–23); *N* = 13 EV-treated condition). *p < 0.05 by Kruskal–Wallis test with multiple comparisons. N refers to independent experiments performed using samples from different donors. **C** EV uptake kinetics. Frequency of pre-activated PBLs positive for CTvio at 1, 4, and 8 h after treatment with CT-Vio-labeled thyTreg-EVs (*N* = 1) with the corresponding EV-associated fraction obtained from culture medium containing CTvio but no cells (Ctrl). **D**–**E** Suppression by thyTreg- and PBL-EVs. Pre-activated PBLs were treated for 3 days with 10⁹, 10⁸, or 10⁷ EV particles derived from thyTregs (*N* = 10) or PBLs (*N* = 4), as well as with SN or SEC protein fractions. Proliferation suppression was expressed as percentage inhibition relative to the control Act + PBLs treated with PBS. Each dot represents one independent experiment. **F** Correlation between target PBL viability (after treatment with 10⁹ thyTreg-EV particles, relative to Act^+^ set at 100%) or the viability of the EV-producing thyTreg on the day of EV isolation and the suppressive capacity of the corresponding EVs. Spearman’s correlation test was used (r = rho). SEC fractions 6–9 were used in these assays

The suppressive ability of thyTreg-EVs on PBL cultures was assessed to determine whether they could, like their parent cells, suppress the proliferation of allogeneic PBL. Activated PBLs (pre-activated for 18 h) were co-cultured for three days with three different amounts of EVs, protein fractions from SEC, or the cleared supernatant of activated thyTreg. The viability of responder CTvio cells (Fig. 2A) was calculated relative to PBS-treated activated CTvio-PBLs, set at 100% (Fig. 2B). Treatment with EVs resulted in variable target cell viability, but no significant differences were observed between the EV conditions (Fig. 2B). In contrast, SEC protein fractions significantly increased the viability of activated PBLs compared to PBS-treated controls (*p* = 0.0129). To assess EV uptake, activated thyTregs were labeled with CTVio. Various volumes of CTVio-labeled EVs, collected after SEC, were added to pre-activated allogeneic responder PBLs, and uptake was evaluated by detecting responder CTVio⁺ PBL via flow cytometry. CTVio⁺ PBLs were detectable as early as 1 h post-incubation, with their frequency correlating with the EV dose tested (Fig. 2C). To exclude the possibility that the CTvio signal resulted from residual free dye, the EV-associated fraction collected after SEC of culture medium alone containing 2µM of CTvio (without cells) was used as a control. No CTvio⁺ PBL were detected, confirming that the CTvio signal observed in Fig. 2C is associated with thyTreg-EVs rather than with free dye.

Functionally, both the highest (10⁹ particles) and intermediate (10⁸ particles) doses of thyTreg-EV-enriched fractions inhibited CD4⁺ and CD8⁺ T cell proliferation with inter-donor variability in the suppressive co-culture assay (Fig. 2D). The lowest dose (10⁷ particles) did not effectively suppress PBL proliferation. As controls, pre-activated CTVio⁺ PBLs were treated with either the cleared supernatant from thyTreg cells without SEC isolation (SN; volume corresponding to the 10⁹ particle EV treatment) or the protein fraction from SEC (fractions 20–23, same volume as 10⁹ particle EVs). Both controls showed a suppressive effect comparable to that of the lowest EV dose (Fig. 2D).

The suppressive function was also tested using EVs derived from activated, non-suppressive PBLs. EVs derived from activated PBLs were included as a functional specificity control to assess whether suppressive and cytotoxic activity are specific to the Treg origin of the vesicles, rather than a generic property of lymphocyte-derived EVs. As expected, none of the SEC-isolated PBL-EV inhibited proliferation. Moreover, PBL-EVs did not induce target cell death as thyTreg-EV-enriched fraction (Supplemental Fig. 2E). In contrast, the cleared supernatant from activated PBLs induced an increase of proliferation in allogeneic responder CTvio^+^ PBLs (Fig. 2E).

Because some thyTreg-EV donors appeared to reduce responder PBL viability (Fig. 2B) and, in some cases, failed to suppress CTvio^+^ PBL proliferation at higher doses (Fig. 2D), we examined the potential correlation between decreased suppressive function and target cell viability following treatment with 10⁹ thyTreg-EV particles (Fig. 2F). A significant negative correlation between responder PBL viability and suppressive capacity suggests that, in some donors, thyTreg-EVs may reduce proliferation, at least in part, through the induction of target cell death, rather than exclusively through classical suppression mechanisms. One possible explanation for this observation is the presence of apoptotic bodies resulting from reduced viability of the EV-producing thyTreg cells. However, no significant correlation was observed between producer cell viability on the day of EV isolation and the suppressive activity of the corresponding EV preparations (Fig. 2F), suggesting that the cytotoxic effect cannot be attributed to reduced viability of the producer cells.

**Fig. 3:**
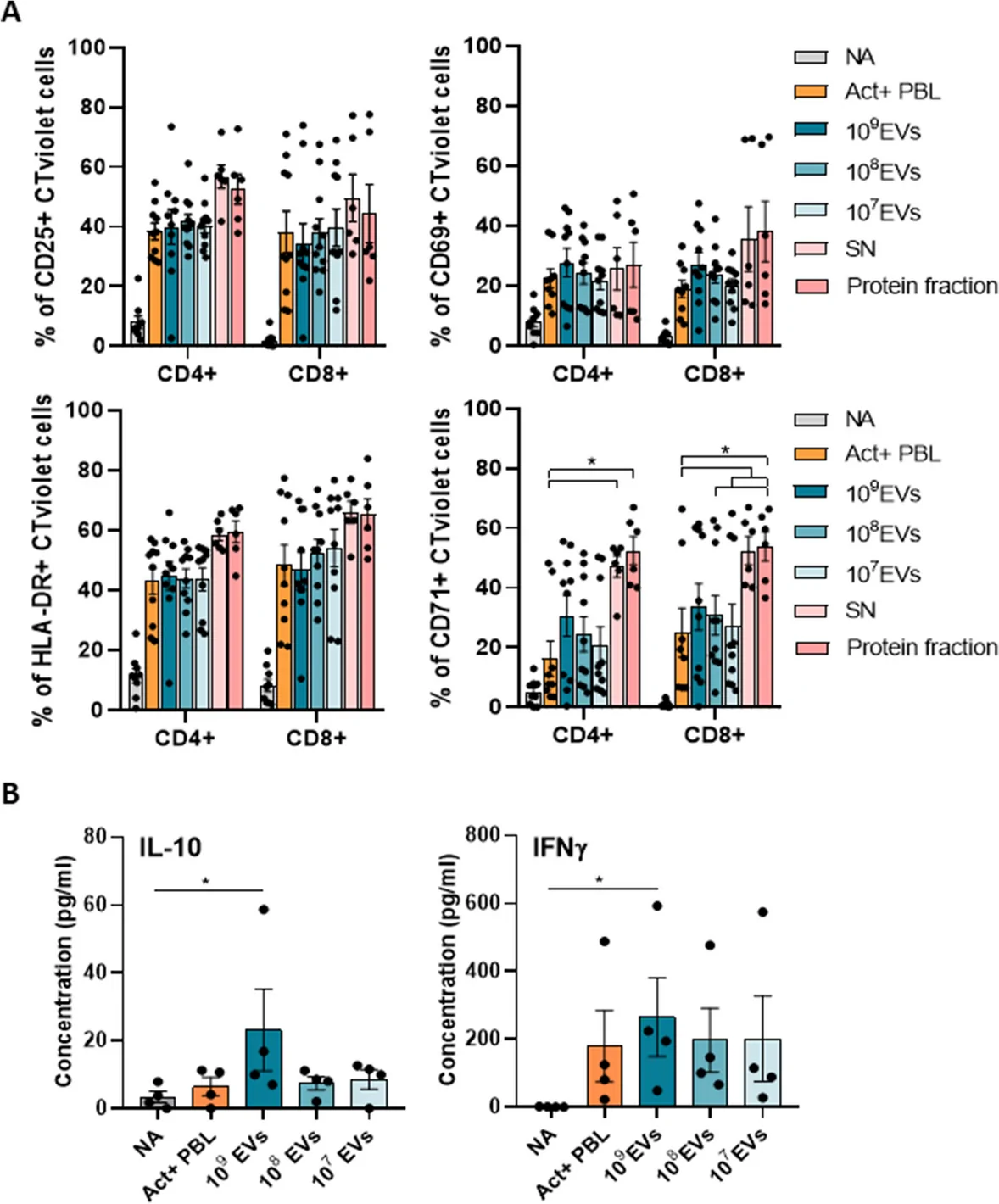
PBL activation markers and cytokine release after thyTreg-EV treatment. **A** Pre-activated PBLs were treated for 3 days with PBS, three doses of thyTreg-EVs (10⁹, 10⁸, 10⁷ particles), cleared supernatant (SN), or SEC protein fractions, then harvested and analyzed by flow cytometry (*N* = 6–9). Fractions 6–9 (EVs) and 20–23 (proteins) from SEC assay were used. Histograms show the frequency of viable PBLs expressing the activation markers CD25, CD69, HLA-DR, and CD71. Each dot represents one independent experiment. *p < 0.05, Kruskal–Wallis test with Dunn’s multiple-comparison correction. **B** Supernatants from non-activated PBLs, activated controls, and pre-activated PBLs treated for 3 days with PBS or thyTreg-particles (10⁹, 10⁸, 10⁷ particles) were collected and analyzed using the ELLA Automated Immunoassay System (*N* = 4). Histograms show IL-10 and IFNγ concentrations (pg/mL). Each point represents one independent experiment. *p < 0.05, two-way ANOVA with Tukey’s post hoc test. NA: non-activated PBLs + PBS; Act^+^: activated PBLs + PBS

We also analyzed activation markers on CTvio^+^ PBLs surface (Supplementary Fig. 3). As expected, the positive control (activated CTvio^+^ PBLs treated with EV-free PBS, Act^+^) showed higher frequencies of activation marker–expressing cells compared to non-activated CTvio^+^ PBLs (NA). None of the thyTreg-EV-enriched doses altered the frequency of CD4⁺ or CD8⁺ activated T-cell marker expression, which remained similar to the CT⁺ control (Fig. 3A). Interestingly, the frequency of CD71⁺ cells was significantly higher following treatment with the cleared supernatant or the protein fraction from SEC (fractions 20–23) compared to Act⁺ PBLs or 10⁹ particles thyTreg-EV treatment.

On the other hand, cytokine production was assessed in the supernatant after 3 days of CTvio^+^ PBL exposure to thyTreg-EVs. Activated PBLs alone (CT⁺) expressed higher levels of IL-10 and IFN-γ compared to CT^−^ (NA), as expected. Despite heterogeneous results, the highest dose of thyTreg-EV-enriched fractions (10⁹ particles) increased both anti-inflammatory (IL-10, *p* = 0.0365) and pro-inflammatory (IFN-γ, *p* = 0.0365) cytokine levels compared to CT^−^ even if not significant (Fig. 3B). In summary, thyTreg-EV-enriched fractions suppressed allogeneic PBL proliferation and induced cell death in some donors but did not significantly affect pre-activated PBL activation markers or cytokine production.

(p) Protein profile characterization of the thyTreg-EVs

**Fig. 4:**
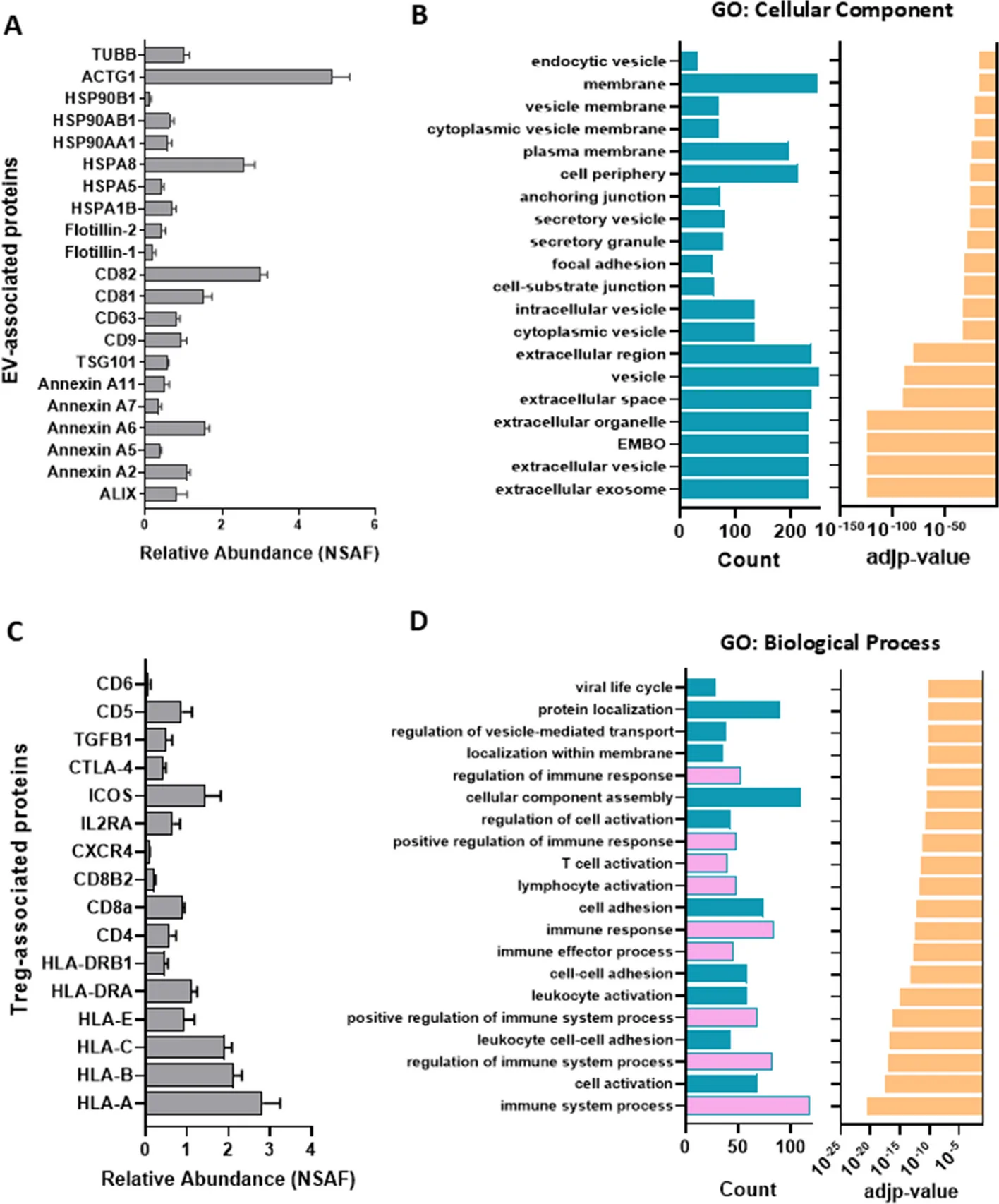
Protein cargo of thyTreg-EVs and Gene Ontology (GO) analysis. The protein cargo of immunocapture-isolated thyTreg-derived EVs was characterized by proteomics to identify proteins associated with EV biogenesis and function. EVs isolated from immunocapture assay. **A** EV-related peptide identification. Average protein relative abundance was calculated using the Normalized Spectral Abundance Factor (NSAF; *N* = 4, mean ± SEM). **B** GO enrichment analysis, Cellular Component domain. Histogram shows the most abundant GO terms enriched in the Cellular Component domain (blue) and their associated adjusted *p*-values (orange, adjp-value), based on proteins only detected in all samples. Count indicates the total number of proteins mapping to each term. **C** Identification of Treg-related classical peptides. Proteomic analysis of thyTreg-EVs (*N* = 4, mean ± SEM) with average protein relative abundance determined by NSAF. **D** GO enrichment analysis. Biological Process domain. Histogram depicts the most abundant biological terms enriched in the Biological Process domain from the GO database (blue and pink) and their associated adjp-value (orange), using only proteins found in all four samples. Count indicates the number of proteins mapping to each GO term

In addition to standard EV characterization, proteomic analysis was performed on tetraspanin-positive thyTreg-EVs immunomagnetically isolated from the culture supernatants of four thyTreg donors. A total of 757 proteins were identified (Supplemental Table 2, proteins sheet), of which 326 remained after exclusion of contaminants. Consistent with prior reports (Tung et al. 2020; Welsh et al. 2024), serum albumin and apolipoproteins were detected as co-isolated contaminants. Because EVs were isolated using immunoglobulins anti-CD9, CD81, and CD63 on immunospheres, all immunoglobulin-related proteins were removed from further analysis. The final dataset therefore consisted of a curated list of 314 proteins (Supplemental Table 2, cleaned). In addition to proteins already known to be associated with EVs (Fig. 4A), we highlighted the 24 most abundant proteins detected across all four samples in Supplemental Fig. 4. Among these, peptidylprolyl cis–trans isomerase B (PPIB), keratin 1 (KRT1), Ras-related protein RAP1A, and syndecan-binding protein (SDCBP) have also previously been implicated in EV biogenesis ()).

Classical EV-associated proteins, including tetraspanins (CD9, CD63, CD81), ALIX, flotillin, annexins, actin, and HSPs, were identified, with CD82, ALIX, HSPA8, and actin among the most abundant (Fig. 4A). The absence of calnexin confirmed the purity of EV preparations and provided partial reassurance against major ER-containing apoptotic body contamination (Supplemental Table 2). The proteomics dataset was further analyzed using Gene Ontology (GO), which revealed that most proteins were associated with various cellular compartments and vesicle types (Fig. 4B, Supplemental Table 3). These findings support the purity of the EVs isolated from thyTreg cultures.

On the other hand, thyTreg-EVs carried proteins characteristic of their parental Treg phenotype (Bernaldo-de-Quirós et al. 2022), including HLA-I, HLA-II, CD25 (IL-2RA), CTLA-4, TGF-β1, and ICOS (Fig. 4C). Moreover, HLA-E, which may contribute to NK cell-mediated cytotoxicity inhibition (Wischhusen et al. 2005), was also detected. Interestingly, EV proteomics also reflected the previously reported co-expression of CD4 and CD8 in thyTregs, which is characteristic of their thymic origin from CD4^+^ CD8^+^ thymocyte precursors. Pathway enrichment analysis revealed a predominance of immune-related processes, such as "immune system process" (GO:0002376), "regulation of immune system process" (GO:0002682), "immune effector process" (GO:0002252), and "Immune Response" (GO:0006955) (Fig. 4D, Supplemental Table 3) highlighting the immunological identity of thyTreg-EVs. In summary, thyTreg-derived EVs displayed canonical EV markers together with Treg-specific proteins, indicating that they retain functional and phenotypic features of their parental cells.

(q) miRNA profile characterization of the thyTreg-EVs.

**Fig. 5:**
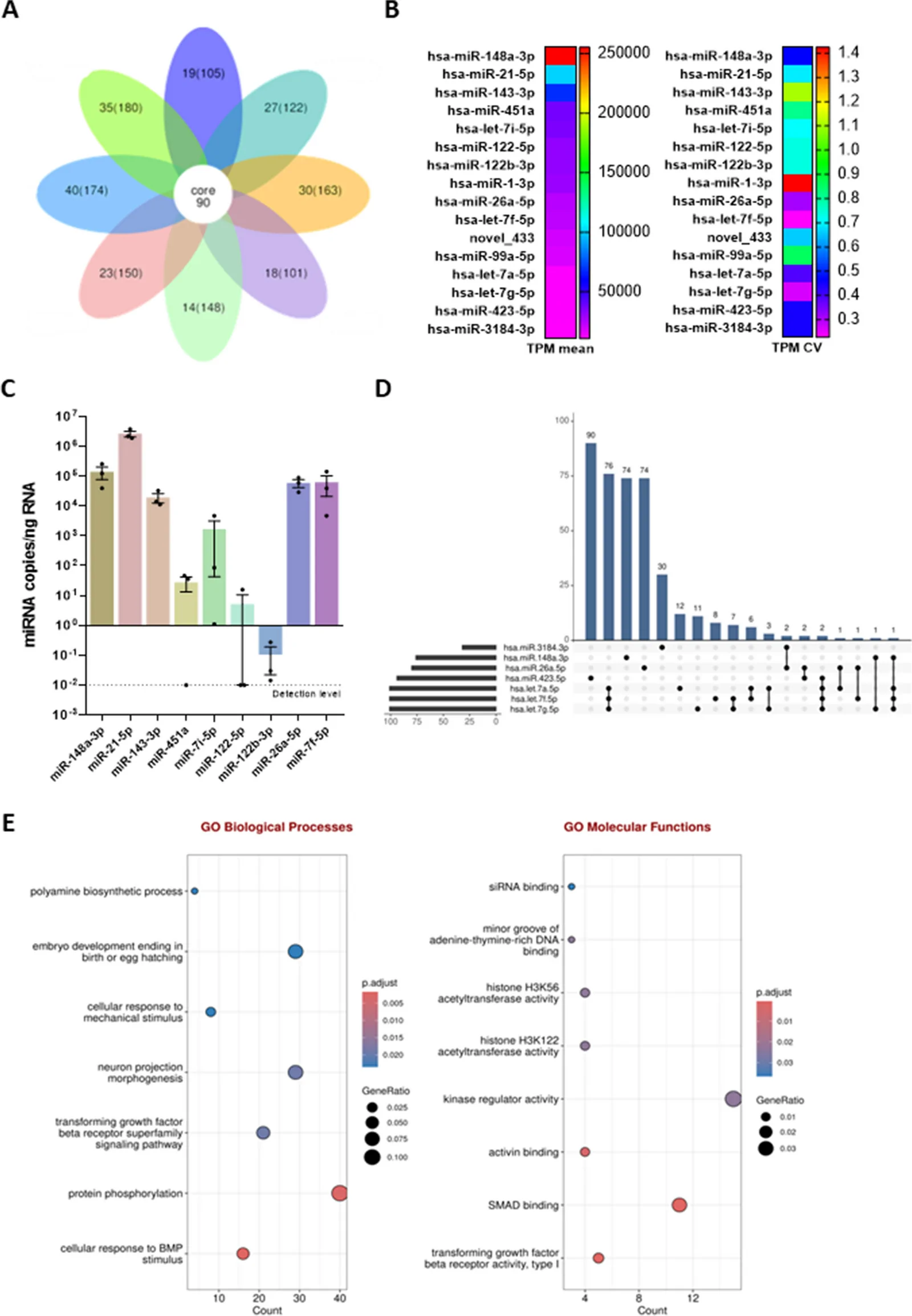
miRNA cargo of thyTreg-EVs and Gene Ontology (GO) analysis. The small RNA profile of immunocapture-isolated thyTreg-derived EVs was analyzed. EVs isolated with immunocapture assay. **A** Venn diagram. Number of miRNAs uniquely detected in each sample; shared miRNAs are indicated in parentheses, and those common to all eight samples are shown in the central area. **B** Highly abundant miRNAs. miRNAs with mean TPM > 10,000 across the 8 samples (left) and their coefficient of variation (CV) of TPM counts, displayed using a color gradient. **C** miRNA quantification by qPCR in ThyTreg cells. Analysis of three independent thyTreg donors. Expression levels were determined using a standard curve generated from miRNA mimics. The dotted line indicates the detection threshold (mean ± SEM). **D** UpSet plot of target gene overlap. Overlap of selected target genes among miRNAs. The lower grid indicates selected miRNAs; the top bar plot shows the number of shared target genes; the bottom-left bar plot shows the total number of target genes within the first decile range for each miRNA. **E** Functional enrichment analysis. GO Biological Processes (left) and Molecular Functions (right) were analyzed using target genes from filtered miRNAs (median TPM > 10,000 and CV < 0.5) within the first decile range. The y-axis shows significantly enriched terms (FDR < 0.05), the x-axis indicates the number of associated target genes, dot color represents adjusted *p*-values (FDR), and circle size reflects the ratio of genes associated with each term relative to the total number of selected target genes

Tetraspanin-positive EVs carry not only proteins but also small RNAs. From eight donor-derived thyTreg-EV samples, deep sequencing identified 554 miRNAs (430 known, 124 novels; Supplemental Table 4), with 90 shared across all samples (Fig. 5A). Statistical analysis of transcripts per million (TPM) counts (Supplemental Table 4) and filtering (mean TPM > 10,000; coefficient of variation (CV) < 0.5) revealed seven highly abundant and stable miRNAs (Fig. 5B). Among them, hsa-miR-148a-3p was the most abundant (mean TPM = 255,151.81; CV = 0.46), while hsa-let-7f-5p was the most consistent (mean TPM = 23,100.41; CV = 0.23). To confirm their presence in parental thyTregs, total RNA was isolated from thyTreg cells of three donors, and several of the most highly expressed EV miRNAs were quantified by real-time PCR (Fig. 5C). With the exception of hsa-miR-451a, hsa-miR-122-5p, and hsa-miR-122b-3p, all thyTreg-EV identified miRNAs were consistently detected in the thyTreg cells.

The seven highly abundant (with a CV < 0.5 and median TPM < 10.000) miRNAs were analyzed using five miRNA-target prediction sources to identify their potential miRNA targets. Iterative analysis of each miRNA yielded a comprehensive list of predicted interactions, although not all miRNAs had annotated targets. The use of multiple libraries enabled ranking of miRNA–target interactions based on scores reflecting predicted negative regulation, which were then aggregated to prioritize target genes (Supplemental Table 5). Given the large number of predictions, we filtered results by retaining genes within the top decile of each ranked list, yielding 401 candidate targets. Figure 5D shows the number of filtered targets per miRNA and their overlaps. Notably, the hsa-let family members (hsa-let-7a-5p, hsa-let-7f-5p, and hsa-let-7 g-5p) shared 76 top-ranked targets. Smaller sets (1–7 genes) were shared by at least two other miRNAs. Each hsa-let miRNA also displayed unique targets: 12, 8, and 11 genes for hsa-let-7a-5p, hsa-let-7f-5p, and hsa-let-7 g-5p, respectively. Likewise, hsa-miR-423-5p, hsa-miR-148a-3p, hsa-miR-26a-5p, and hsa-miR-3184-3p exhibited 90, 74, 74, and 30 unique targets, respectively.

GO-based enrichment identified seven biological processes, and eight molecular functions significantly associated with the predicted target genes (Fig. 5E, Supplemental Table 6). The enriched biological processes with the greatest number of associated target genes were protein phosphorylation (GO:0006468; 40 genes) and neuron projection morphogenesis (GO:0048812; 29 genes). Several enriched molecular function terms were related to TGF-β receptor signaling and activity, including SMAD binding (GO:0046332), transforming growth factor beta receptor activity type I (GO:0005025), and activin binding (GO:0048185). Notably, GO terms directly linked to TGF-β signaling, such as cellular response to Bone Morphogenetic Protein (BMP) stimulus (GO:0071773) and SMAD binding, showed the lowest FDR values and involved the largest number of genes. Key genes, including *TGFBR1*, members of the activin A receptor family, and Ski-like protein (*SKIL*), were annotated to five or more GO terms across both biological processes and molecular functions (Supplemental Fig. 5, Supplemental Table 6). Further functional enrichment analysis was performed using the KEGG pathway database. The MAPK signaling pathway (hsa04010) and pluripotency stem cell–related signaling pathways (hsa04550) were significantly enriched, with 12 and 16 associated target genes, respectively (Supplemental Table 6).

As shown in Fig. 5D, the miRNA family hsa-let-7a-5p/7f-5p/7g-5p exhibited a high proportion of shared target genes. Genes, such as *RICTOR*, *EZH2*, and *ABL2*, were specifically targeted by the miRNA pairs hsa-let-7g-5p/hsa-miR-148a-3p, hsa-let-7f-5p/hsa-miR-26a-5p, and hsa-let-7a-5p/hsa-miR-26a-5p, respectively (Supplemental Fig. 5). The enriched functions associated with these three genes included protein phosphorylation (GO:0006468), embryo development ending in birth or egg hatching (GO:0009792), and kinase regulator activity (GO:0019207).

**Fig. 6:**
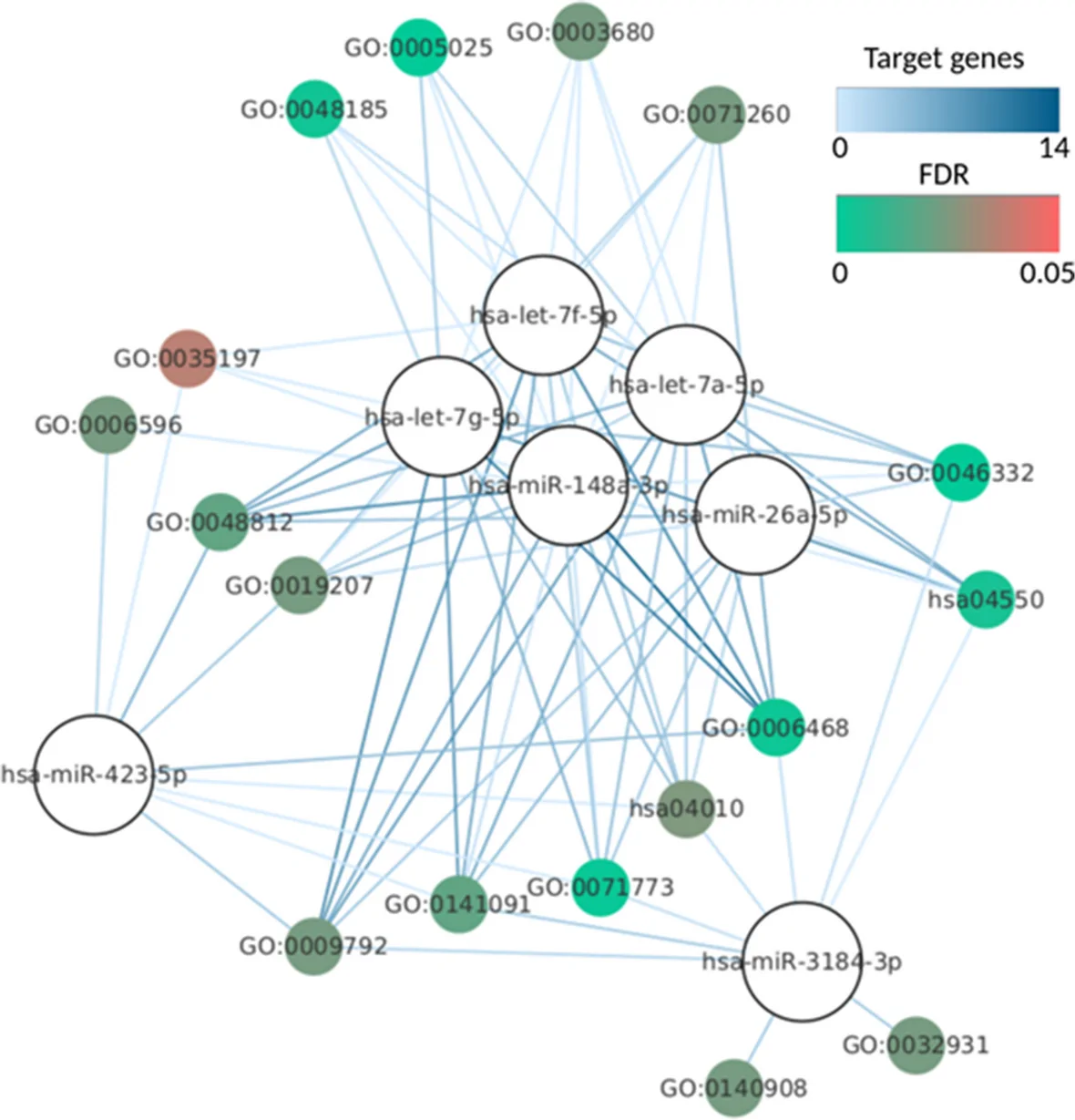
Network of miRNAs and GO Biological Process and Molecular Function associations of thyTreg-particles. EVs isolated with immunocapture assay. The network shows associations (edges) between miRNAs (white nodes with black borders) and enriched functions (nodes colored green to red). Edge color (blue gradient) reflects the number of selected target genes contributing to each association. Node color indicates FDR significance from the functional enrichment analysis, with green-to-red representing increasing significance

A network analysis was then constructed to visualize the relationships between miRNAs and their enriched functions, with edge gradients representing the number of target genes supporting each association (Fig. 6). A cluster, including activin binding (GO:0048185), TGF-β receptor activity type I (GO:0005025), minor groove adenine–thymine-rich DNA binding (GO:0003680), and cellular response to BMP stimulus (GO:0071260), was associated with the group of five miRNAs (hsa-let-7a-5p, hsa-let-7f-5p, hsa-let-7g-5p, hsa-miR-148a-3p, hsa-miR-26a-5p), but not with hsa-miR-423-5p or hsa-miR-3184-3p. Another group of enriched functions, including siRNA binding (GO:0035197), polyamine biosynthetic process (GO:0006596), neuron projection morphogenesis (GO:0048812), and kinase regulator activity (GO:0019207), was shared by the hsa-let-7 family members, hsa-miR-148a-3p, hsa-miR-26a-5p, and hsa-miR-423-5p. The molecular functions histones H3K56 and H3K122 acetyltransferase activities (GO:0140908 and GO:0032931) were uniquely associated to hsa-miR-3184-3p. On the other hand, some groups of enriched functions were associated not only with the five previously mentioned miRNAs, but also specifically with one of the two remaining miRNAs (hsa-miR-423-5p and hsa-miR-3184-3p). For instance, the groups comprising SMAD binding (GO:0046332) and signaling pathways regulating pluripotency of stem cells (hsa04550) were also associated with hsa-miR-3184-3p. In addition, some significantly enriched functions were linked to a greater number of selected target genes and were associated with the seven studied miRNAs, including protein phosphorylation (GO:0006468), MAPK signaling pathway (hsa04010), cellular response to BMP stimulus (GO:0071773), transforming growth factor beta receptor superfamily signaling pathway (GO:0141091), and embryo development ending in birth or egg hatching (GO:0009792) (Fig. 6, Supplemental Table 6). In summary, the enriched GO terms converge on pathways that integrate signaling regulation, epigenetic modification, and metabolic activity to sustain cell proliferation and differentiation. In particular, functions related to TGF-β/BMP signaling (GO:0071773, GO:0141091, GO:0046332, GO:0005025, GO:0048185) and protein phosphorylation and kinase regulation (GO:0006468, GO:0019207) highlight the central role of signal transduction in controlling growth responses. At the chromatin level, histone acetyltransferase activities (GO:0032931, GO:0140908) and DNA/RNA binding (GO:0003680, GO:0035197) point to transcriptional and post-transcriptional mechanisms that fine-tune gene expression during proliferation.

## Discussion

In the present study, CD25^+^ FOXP3^+^ thyTreg were shown to produce EVs with suppressive functions, as evidenced by their ability to inhibit target cell proliferation. It is important to note that the isolation strategy employed in this study (SEC and immunocapture) selectively enriches for EVs and does not capture the full spectrum of EV subtypes released by thyTregs. Previous studies characterizing EVs derived from human peripheral blood Tregs reported mean sizes of approximately 150 nm and modes of approximately 125 nm by NTA (Tung et al. 2020; Torri et al. 2017). These values are smaller than those observed in our thyTreg-derived preparations (mean ~ 212 nm, mode ~ 183 nm). This difference between our results and those previously published may reflect the distinct cellular origin (peripheral blood versus thymus) and/or the different isolation methods employed across studies, which include ultracentrifugation, ExoSpin, the ExoMiR commercial kit, differential ultrafiltration combined with SEC, and immunocapture. EV isolation method is a well-recognized determinant of the size distribution of recovered particles (Welsh et al. 2024). Similar suppressive effects of Treg-derived EVs have been described in murine models, where they inhibit CD8⁺ T cell proliferation via cell cycle arrest at G0/G1 and induction of cell death (Chen et al. 2019). However, the underlying mechanisms remain incompletely defined. In mice, one proposed mechanism involves CD73 expression on EVs (Smyth et al. 2013), but in our study neither thyTregs nor their EVs expressed CD73, suggesting this pathway is not relevant in human thyTreg-EVs. Although CTLA-4 is often linked to Treg suppression, blocking CTLA-4 on murine EVs did not affect their function (Smyth et al. 2013), however human thyTreg-EVs express CTLA-4. It would be interesting to investigate whether human CTLA-4 expressed on EVs retains functional activity. CD25 may contribute by depleting local IL-2 (Tung et al. 2020; Smyth et al. 2013), which could be consistent with the high CD25 (IL2RA) levels detected in thyTreg-EVs, suggesting that IL-2 depletion may represent one of the mechanisms underlying their suppressive activity. The detection of CD8 in thyTreg-EV proteomics is consistent with the known co-expression of CD4 and CD8 in thymic Tregs, which retain features of their double-positive thymocyte precursor origin, as previously described for this cell population (Bernaldo-de-Quirós et al. 2022). Proteomic screening also revealed that thyTreg-EVs contained TGF-β1, a key immunoregulatory cytokine known to confer suppressive functions on conventional T cells (Wang et al. 2023), whereas IL-10 was not detected. Thus, TGF-β1 carried by thyTreg-EVs may represent another suppressive mechanism since conventional T cells exposed to TGF-β1 can acquire regulatory phenotypes and functions (Liu et al. 2018; Strauss et al. 2008). In addition, ICOS was identified, a molecule implicated in multiple immune functions. Notably, ICOS⁺ EVs together with checkpoint inhibitors and TGF-β1 have been reported to induce immunosuppression and convert CD4⁺CD25⁻ T cells into IL-10–producing type 1 regulatory T cells (Solinas, Gu-Trantien, Willard-Gallo 2020; Campos-Mora et al. 2022), indicating another potential mechanism of action for thyTreg-EVs. These proposed mechanisms of action for thyTreg-EVs warrant further investigation in future studies. A limitation of this study is that the proteomic analysis was performed on thyTreg-EVs isolated using anti-CD81, CD9, and CD63 magnetic beads, meaning we cannot exclude the possibility that proteins from CD81⁻CD9⁻CD63⁻ EV subpopulations may also contribute to suppressive mechanisms.

A major observation in this study was that thyTreg-EV-enriched fractions derived from some, but not all, donors suppressed PBL proliferation and induced cell death, but only modestly affecting activation marker expression. The distinction between cytotoxicity and classical immunosuppression is relevant here: while both reduce target cell numbers, they operate through distinct mechanisms. At the highest EV dose tested (10⁹ particles), a reduction in responder PBL viability was observed in a subset of donors. While non-specific toxicity from particle overload cannot be fully excluded, the donor-dependence of this effect argues against a purely non-specific mechanism. The inter-donor variability in thyTreg-EV suppressive capacity warrants consideration. Several factors may contribute to the observed variability: differences in thyTreg activation state and EV biogenesis efficiency across donors could affect cargo composition, exemplified by the detection of granzyme B in only 3 of 4 proteomic samples; the sensitivity of responder PBLs to EV-mediated suppression; and the fact that thymic tissue sources introduce inherent biological variability related to donor age, diagnosis, and immune history. However, apoptotic markers were not assessed on SEC-purified EVs, and the absence of calnexin in the immunocapture-purified proteomic dataset cannot be extrapolated to the SEC-purified preparations used functionally. Future studies should include apoptotic marker assessment on SEC-purified EVs. Identifying the molecular determinants of suppressive potency, for example, through correlation of EV cargo composition with functional outcomes across donors, will be an important goal for future studies aimed at standardizing thyTreg-EV preparations for clinical use. One limitation of this study is that such correlation was not possible because the available isolated-EV material had to be allocated either to proteomic/miRNA analyses or to functional assays. PBL death could be mediated by granzyme B, which was detected in 3 out thyTreg-EV samples by proteomic analysis and has also been identified as a key mediator of Treg-EV suppressive function in murine models (Campos-Mora et al. 2022). It should be noted that PBLs were activated 18 h prior to EV treatment. During the first 24 h of stimulation, cells are generally preparing for clonal expansion but are not yet actively proliferating (Weerakoon et al. 2024; Kaech et al. 2002). Thus, administering EVs after 18 h may have had only a limited impact on cellular activation, which was likely already initiated. By contrast, suppressive effects might be more evident during the proliferation phase, which typically begins after 24 h. Therefore, the observed differences between activation and proliferation outcomes likely reflect the sequential progression of these processes rather than distinct effects of thyTreg-EV-enriched fractions on activation versus proliferation.

One of the most intriguing findings was the increase in nearly all tested cytokines in the supernatant of activated PBL cultures treated with the highest dose of thyTreg-EVs. This included elevated levels of IL-10, a cytokine central to classical Treg-mediated suppression, as well as IFN-γ. Importantly, these cytokines were not detected in thyTreg-EVs by proteomic screening (Supplemental Table 2), indicating that their presence likely reflects target cell responses to EV treatment rather than direct transfer. This observation is limited by the small number of replicates, preventing robust statistical conclusions, and should therefore be validated in future studies. It will also be important to determine whether the concurrent increase in both anti- and proinflammatory cytokines influences cell viability or function beyond the three-day culture period. The combination of proliferation inhibition and cytokine elevation may contribute to subsequent cell death or cellular dysfunction.

In addition to their protein cargo, thyTreg-derived EVs were enriched in a distinct repertoire of small RNAs, including a set of highly abundant and stable miRNAs shared across donors. While SEC effectively removes free RNA and RNA–protein complexes, because miRNA profiling was performed on immunocapture-purified rather than SEC-purified EVs, we cannot exclude the possibility that the miRNA signature reported here partially reflects apoptotic body-derived vesicles rather than exclusively bona fide EVs. Members of the hsa-let-7 family and hsa-miR-148a-3p dominated the EV miRNA landscape, suggesting selective packaging rather than passive incorporation. The predicted target profiles of these miRNAs converged on pathways central to signal transduction, particularly TGF-β/BMP signaling and MAPK-mediated phosphorylation cascades, as well as kinase regulation and chromatin remodeling. These findings are consistent with the established role of TGF-β signaling in immune regulation and Treg stability (Liu et al. 2018), raising the possibility that thyTreg-EVs may reinforce tolerogenic programs in recipient cells through coordinated post-transcriptional modulation of key signaling nodes. The enrichment of targets involved in pluripotency-associated pathways and epigenetic regulation further suggests a capacity to influence cellular differentiation and proliferative responses. Together, these data support a model in which thyTreg-EVs deliver a structured miRNA cargo capable of reshaping signaling networks linked to proliferation, differentiation, and immune regulation, thereby contributing to their observed suppressive activity.

In this study, we demonstrated for the first time that human thyTregs release EVs, which we comprehensively characterized by proteomics and transcriptomics. We cannot exclude a contribution of co-isolated soluble factors to the observed functional effects, as the cleared supernatant also showed suppressive activity. Future studies using EV-depleted supernatants or EV inactivation controls (such as proteinase K treatment, freeze–thaw disruption) will be needed to definitively attribute the observed effects to EVs. Our findings also suggest that the mechanisms of human thyTreg-EV-enriched fractions differ from those described in murine models, particularly in their capacity to inhibit lymphocyte proliferation. From a translational perspective, tetraspanin-positive thyTreg-derived EVs represent a cell-free alternative to Treg cell therapy. Unlike living cells, EVs offer greater stability, scalability, and the potential for off-the-shelf allogeneic use. The ongoing clinical trial NCT04924491, which demonstrated the safety of thyTreg infusion in pediatric heart transplant recipients, provides a clinical framework within which EV-based strategies could be developed. Potential indications include solid organ transplantation, graft-versus-host disease, and autoimmune diseases, in which Treg-based immunomodulation has shown promise. Current evidence suggests that extracellular vesicles exhibit relatively low baseline immunogenicity and are generally well tolerated compared with cell-based therapies. Nevertheless, EVs are not immunologically inert; their immunogenicity depends on their cellular origin, surface composition, cargo, and recipient characteristics (Driedonks et al. 2022; Prunevieille et al. 2021). Therefore, the immunogenicity of thyTreg-EVs remains an important consideration and should be addressed in future in vivo studies before clinical translation. Overall, these findings lay the groundwork for future preclinical and clinical evaluation of thyTreg-EVs as immunomodulatory agents.

## Conclusions

This study provides the first evidence that human thymic Treg cells (thyTregs) produce EVs with donor-variable immunosuppressive activity. These EVs inhibited peripheral blood lymphocyte proliferation with inter-donor variability and, in some cases, induced cell death. Proteomic profiling revealed a distinctive cargo enriched in immunoregulatory proteins, including CD25, CTLA-4, TGF-β1, ICOS, and granzyme B, supporting multiple potential mechanisms of action. Notably, the absence of CD73 and IL-10, which are commonly implicated in murine Treg-EV function, underscores fundamental differences between human and mouse models and highlights the need for human-specific studies. These findings establish thyTreg-derived EVs as a novel, cell-free modality with potential for immune modulation and treatment of immune-mediated diseases. By advancing our understanding of their composition and function, this work lays the foundation for EV-based immunotherapies offering improved stability, safety, and scalability compared with conventional cell-based approaches.

## Supplementary Information

Supplementary Material 1. Supplemental Figure 1: Suppressive function of thyTreg cells on allogeneic CTVio-labeled PBLs. Representative histograms showing CellTrace Violet dilution in responder PBLs, indicating cell proliferation, gated on viable CD4⁺ or CD8⁺ T cells. NA: non-activated PBLs. Supplemental Figure 2: Characteristics of activated PBLs. Representative dot plot of FOXP3⁺ and CD25⁺ cells, gated on live cells. Histogram showing viability frequencies of activated PBLs on the day of EV isolation. Histogram of Treg frequencies in activated PBLs on the day of EV isolation. Suppressive capacity of activated PBLs on allogeneic CTVio-labeled PBLs. Suppression of CD4⁺ and CD8⁺ responder T cell proliferation in coculture with activated allogeneic PBLs, calculated using the division index method. Supplemental Figure 3: Activation marker expression on responder CTVio-labeled PBLs. Representative overlay histograms showing the expression of CD25, CD69, HLA-DR, and CD71 on non-activated PBLsand activated PBLs, gated on viable CD8⁺ T cells. Supplemental Figure 4: Most abundant proteins in thyTreg-EVs. The 24 most abundant proteins across all four samples are shown. Average protein relative abundance was calculated using the Normalized Spectral Abundance Factor. Supplemental Figure 5: Gene associations with GO terms and miRNAs. Heatmap showing clustering of filtered target genesand enriched functionsbased on the presence of associations. Dendrograms indicate clustered gene setsand enriched functions. The bar annotation on the right represents FDR values for enriched functions. Each row of the grid above the heatmap shows the final rank values of associated target genes for each miRNA

Supplementary Material 2. Supplemental Table 1: Antibodies used in Flow Cytometry and Western Blot. Upper panel: Antibodies used to define ThyTreg cell phenotype. For each antibody, the fluorochrome is indicated. Lower Middle panel: Antibodies used to analyze PBL viability, proliferation and activation state after a 3-day co-culture with or without ThyTreg or ThyTreg-EVs. Lower panel: Primary and secondary antibodies used in Western Blot. MTY: Miltenyi Biotec. BC: Beckman Coulter. BD: Beckton Dickinson. TFS: ThermoFisher Scientific. IS: ImmunoStep.

Supplementary Material 3. Supplemental Table 2: Peptides and proteins identified by proteomic analysis. Peptide identification in thyTreg-EVs. PSM: Peptide-Spectrum Matches. MH+ [Da]: Theoretical Molecular Ion [Dalton]. m/z [Da]: mass-to-charge ratio [Daltons]. Deltam/z [Da]: change in mass-to-charge ratioof an ion compared to a baseline value. RT [min]: Retention Time [minutes]. AAs: amino acids. MW [kDa]: Molecular Weight [kilodaltons]. calc. pI: calculated isoelectric point. # PSMs: Number of Peptide-Spectrum Matches. NSAF: Normalized Spectral Abundance Factor.

Supplementary Material 4. Supplemental Table 3: Identified Biological Processes. Biological term enrichment was performed using the following databases: Gene Ontology, Reactome, WikiPathways, Human Protein Atlas, and CORUM. Category: Database source of the term. adj.P.Val: Adjusted *p*-value after correction for multiple testing. Term_size: Total number of proteins annotated in the term. Effective_domain_size: List of protein identifiers mapping to the term. geneID: Numbers of protein identifiers used for the hypergeometric test. Gene Ratio: Count/query_size. Bg ratio: term_size/geneID.

Supplementary Material 5. Supplemental Table 4: Small RNAs detected in thyTreg-EVs. Hsa-miR and novel small RNA detection in thyTreg-EVs. Read count: the number of sequencing reads mapping to each small RNA, reflecting its abundance. TPM: normalizes read counts for sequencing depth, reflecting relative small RNA abundance. SD: standard deviation. CV: coefficient of variation.

Supplementary Material 6. Supplemental Table 5: Determination of RNA targets. Determination of DNA targets for seven specific miRNAs, hsa-let-7a-5p, hsa-let-7f-5p, hsa-let-7g-5p, hsa-miR-148a-3p, hsa-miR-26a-5p, hsa-miR-3184-3p, and hsa-miR-423-5p, using algorithmsfor target specificity. EntrezID: gene identifier of the target sequence. Number: rank assigned by each algorithm, with the final mean rank indicating the most likely targets for each miRNA.

Supplementary Material 7. Supplemental Table 6: Identified Biological Processes for miRNA targets. GO Biological Processes, GO Molecular Functions, and KEGG pathways significantly associated with miRNA targets. GOID: identifier of the GO term corresponding to its GO name. The table also lists the genes included in each determined pathway.

## Data Availability

All data supporting the findings of this study are available within the paper and its Supplementary Information.

## Abbreviations

7-AAD: 7-Aminoactinomycin D
ABL2: Abelson Tyrosine-Protein Kinase 2
ALIX: ALG-2-interacting protein X
ATP: Adenosine triphosphate
BCA: Bicinchoninic Acid
BMP: Bone Morphogenetic Protein
BP: Biological Process
CC: Cellular Component
cDNA: Complementary Deoxyribonucleic Acid
CT-: Negative control
CT +: Positive control
CTLA-4: Cytotoxic T-lymphocyte associated protein 4
CTvio: CellTrace Violet
CV: Coefficient of variation
DDA: Data-dependent acquisition
DNA: Deoxyribonucleic Acid
ELLA: Ella Automated Immunoassay System
EM: Electron microscopy
EVs: Extracellular vesicles
EZH2: Enhancer of Zeste Homolog 2
FBS: Fetal bovine serum
FDR: False discovery rate
FMO: Fluorescence minus one
FOXP3: Forkhead box P3
GMP: Good Manufacturing Practice
GO: Gene Ontology
GvHD: Graft-versus-host disease
H3K56: Histone H3 Lysine 56
H3K122: Histone H3 Lysine 122
HCD: High Collision Dissociation
HGUGM: Hospital General Universitario Gregorio Marañón
HLA: Human Leukocyte Antigen
HPA: Human Protein Atlas
HPLC: High-Performance Liquid Chromatography
HRP: Horseradish peroxidase
HSA: Homo sapiens
HSP: Heat shock protein
ICOS: Inducible T-cell COStimulator
ICTS-CNME: National Centre of Electron Microscopy in Madrid
IFNγ: Interferon gamma
IL: Interleukin
IL-2RA: Interleukin-2 Receptor Alpha
KEGG: Kyoto Encyclopedia of Genes and Genomes
KRT1: Keratin 1
LAG-3: Lymphocyte-activation gene 3
LOD: Limit of detection
MAPK: Mitogen-Activated Protein Kinase
MF: Molecular Function
miR: MiRNA: microRNA
MS: Mass Spectrometry
NK: Natural killer
NSAF: Normalized Spectral Abundance Factor
NTA: Nanoparticle Tracking Analysis
PBLs: Peripheral blood lymphocytes
PBL-EVs: Extracellular vesicles derived from PBLs
PBMC: Peripheral blood mononuclear cells
PBS: Phosphate-Buffered Saline
PCR: Polymerase Chain Reaction
PD-1: Programmed cell death protein 1
PPIB: Peptidylprolyl cis–trans isomerase B
PSM: Peptide-Spectrum Matches
pTregs: Peripheral regulatory T cells
PVDF: Polyvinylidene fluoride
qPCR: Quantitative real-time PCR
RAP1A: Ras-related protein, isoform 1A
REAC: Reactome
RICTOR: Rapamycin-Insensitive Companion of mTOR
RNA: Ribonucleic Acid
mRNA: Messenger RNA
rRNA: Ribosomal RNA
snRNA: Small nuclear RNA
snoRNA: Small nucleolar RNA
tRNA: Transfer RNA
RPC: Reversed-phase chromatography
RPMI: Roswell Park Memorial Institute medium
SDCBP: Syndecan-binding protein
SEC: Size exclusion chromatography
SEM: Standard Error of the Mean
SKIL: Sloan-Kettering Institute-like protein
SN: Supernatant
Tconv: Conventional T cells
TCR: T cell receptor
TIGIT: T cell immunoreceptor with Ig and ITIM domains
TGF-β: Transforming growth factor-beta
TGFBR1: Transforming Growth Factor Beta Receptor 1
thyTregs: Tregs isolated from human thymic tissue
thyTreg-EVs: Extracellular vesicles derived from human thymic tissue
TNFα: Tumour Necrosis Factor alpha
TPM: Transcripts per million
Tregs: Regulatory T cells
TSG101: Tumor Suppressor Gene 101
tTregs: Peripheral thymus-derived Treg
U/mL: Units per milliliter
WP: WikiPathways
