# Supplementary material for: Human thymic Treg-derived extracellular vesicles display a defined proteomic and miRNA signature with donor-variable suppressive activity
Source: Mol Med. 2026 Aug 6;32:201. doi: 10.1186/s10020-026-01576-4 (PMC13729346; doi:10.1186/s10020-026-01576-4)
Supplement: Supplementary file 1 [file 10020_2026_1576_MOESM2_ESM.docx]

**Supplemental Table 1**

| ThyTreg cell phenotype | | | |
| --- | --- | --- | --- |
| **Antibody** | **Fluorescence** | **Clone** | **Supplier** |
| CD3 | VioGreen | REA613 \| SK7 | MTY |
| CD73 | BV605 | AD2 | Biolegend |
| LAG-3 | BV650 | 11C3C65 | Biolegend |
| CD39 | FITC | MZ18-23C8 | MTY |
| CD45RA | ECD | 2H4LDH11LDB9 | BC |
| HLA-DR | PC5.5 | Immu-357 | BC |
| CD25 | PE-Cyanine7 | BC96 | eBioscience |
| CD8 | APC-A700 | B9.11 | BC |
| CD4 | APC-A750 | 13B8.2 | BC |
| Viability dye | eFluor 450 | - | eBioscience |
| CTLA-4 | APC | BNI3 | BD |
| FOXP3 | PE | PCH101 | eBioscience |

| PBL viability, proliferation, and activation state | | | |
| --- | --- | --- | --- |
| **Antibody** | **Fluorescence** | **Clone** | **Supplier** |
| Proliferation dye | Cell Trace violet | - | TFS |
| CD3 | VioGreen | REA613 \| SK7 | MTY |
| CD8a | BV570 | RPA-T8 | Biolegend |
| CD73 | BV605 | AD2 | Biolegend |
| CD4 | PE-Vio615 | REA623 \| SK3 | MTY |
| Viability dye | 7AAD | - | Merck |
| CD25 | PE-Cyanine7 | BC96 | eBioscience |
| HLA-DR | APC | GRB-1 | IS |
| CD69 | Alexa Fluor700 | FN50 | Biolegend |
| CD71 | APC-Vio770 | REA902 \| AC102 | MTY |

| Western Blot | | |
| --- | --- | --- |
| **Antibody** | **Supplier** | **Clone** |
| CD9 | Abcam | polyclonal |
| TSG101 | Santa Cruz Biotechnology | tsg 101 (C-2) |
| donkey anti-rabbit IgG | GE Healthcare | polyclonal |
| sheep anti-mouse IgG | GE Healthcare | polyclonal |

**Supplemental Table 1: Antibodies used in Flow Cytometry and Western Blot.** Upper panel: Antibodies used to define ThyTreg cell phenotype. For each antibody, the fluorochrome is indicated. Middle panel: Antibodies used to analyze PBL viability, proliferation and activation state after a 3-day co-culture with or without ThyTreg or ThyTreg-EVs. Lower panel: Primary and secondary antibodies used in Western Blot. MTY: Miltenyi Biotec. BC: Beckman Coulter. BD: Beckton Dickinson. TFS: ThermoFisher Scientific. IS: ImmunoStep.
